# 加速期慢性淋巴细胞白血病患者的临床和分子生物学特征分析

**DOI:** 10.3760/cma.j.issn.0253-2727.2023.11.006

**Published:** 2023-11

**Authors:** 子元 周, 罗梦佳 戴, 业钦 沙, 彤璐 邱, 姝超 秦, 祎 缪, 奕 夏, 微 吴, 翰宁 唐, 卫 徐, 建勇 李, 华渊 朱

**Affiliations:** 南京医科大学第一附属医院，江苏省人民医院血液科，南京 210029 Department of Hematology, The First Affiliated Hospital of Nanjing Medical University, Jiangsu Province Hospital, Nanjing 210029, China

**Keywords:** 白血病，淋巴细胞，慢性，B细胞, 正电子发射断层显像计算机体层摄影术, 分子生物学, 预后, Leukemia, lymphocytic, chronic, B-cell, Positron emission tomography computed tomography, Molecular biology, Prognosis

## Abstract

**目的:**

探索加速期慢性淋巴细胞白血病（aCLL）患者的临床和分子生物学特征。

**方法:**

回顾性分析2020年1月至2022年10月在南京医科大学第一附属医院血液科诊断的13例aCLL患者的临床资料，分析aCLL患者的临床及分子生物学特征。

**结果:**

13例患者诊断aCLL时的中位年龄为54（35～72）岁。5例患者既往未治疗，8例患者接受以布鲁顿酪氨酸激酶抑制剂（BTKi）为主的治疗出现疾病进展时通过病理确诊aCLL，6例存在大包块病灶（病灶最大径≥5 cm）。PET-CT显示病灶间及同一病灶内部代谢摄取存在异质性，其中代谢摄取最高处病灶的中位最大标准摄取值（SUVmax）为6.96（2.51～11.90）。免疫球蛋白重链可变区（IGHV）无突变患者占76.9％（10/13），常见的遗传学及分子学异常包括+12（42.9％，3/7）、ATM突变（50％，6/12）、NOTCH1突变（50％，6/12）。12例患者接受后续治疗，总反应率为91.7％，完全缓解率为58.3％，5例患者出现疾病进展，其中2例患者发生Richter转化，伴KRAS突变的aCLL患者无进展生存时间显著缩短（7.0个月对26.3个月，*P*＝0.015）。

**结论:**

aCLL患者临床呈侵袭性，多合并预后不良因素（IGHV无突变、+12、ATM突变、NOTCH1突变等），临床怀疑疾病进展（特别是大包块病灶）且SUVmax≥5的患者在代谢摄取最高部位行活检以明确病理诊断。

## Abstract (en)

**Objective:**

To investigate the clinical and molecular biological characteristics of patients with accelerated chronic lymphocytic leukemia (aCLL).

**Methods:**

From January 2020 to October 2022, the data of 13 patients diagnosed with aCLL at The First Affiliated Hospital of Nanjing Medical University were retrospectively analyzed to explore the clinical and molecular biological characteristics of aCLL.

**Results:**

The median age of the patients was 54 (35–72) years. Prior to aCLL, five patients received no treatment for CLL/small lymphocytic lymphoma (SLL), while the other patients received treatment, predominantly with BTK inhibitors. The patients were diagnosed with aCLL through pathological confirmation upon disease progression. Six patients exhibited bulky disease (lesions with a maximum diameter ≥5 cm). Positron emission tomography (PET) -computed tomography (CT) images revealed metabolic heterogeneity, both between and within lesions, and the median maximum standardized uptake value (SUVmax) of the lesion with the most elevated metabolic activity was 6.96 (2.51–11.90). Patients with unmutated IGHV CLL accounted for 76.9％ (10/13), and the most frequent genetic and molecular aberrations included +12 [3/7 (42.9％)], ATM mutation [6/12 (50％)], and NOTCH1 mutation [6/12 (50％)]. Twelve patients received subsequent treatment. The overall response rate was 91.7％, and the complete response rate was 58.3％. Five patients experienced disease progression, among which two patients developed Richter transformation. Patients with aCLL with KRAS mutation had worse progression-free survival (7.0 month *vs* 26.3 months, *P*＝0.015).

**Conclusion:**

Patients with aCLL exhibited a clinically aggressive course, often accompanied by unfavorable prognostic factors, including unmutated IGHV, +12, ATM mutation, and NOTCH1 mutation. Patients with CLL/SLL with clinical suspicion of disease progression, especially those with bulky disease and PET-CT SUVmax ≥5, should undergo biopsy at the site of highest metabolic uptake to establish a definitive pathological diagnosis.

加速期慢性淋巴细胞白血病（aCLL）定义为慢性淋巴细胞白血病（CLL）/小淋巴细胞淋巴瘤（SLL）的组织病理中存在增殖中心扩张或融合（大于20倍高倍视野），且Ki-67>40％或每个增殖中心>2.4个有丝分裂象，是CLL/SLL发生组织学进展的一种特殊病理类型[1]–[2]。既往报道提示，aCLL临床呈侵袭性且预后不佳，多表现为LDH高水平、ZAP-70高表达、PET-CT的最大标准摄取值（SUVmax）高于其他CLL/SLL以及易合并17p−、11q−等不良预后因素，其总生存（OS）时间显著缩短[2]–[4]。本研究纳入了13例在我中心诊断的aCLL患者，回顾性分析其临床及分子生物学特征。

## 病例与方法

1. 病例：本研究纳入了2020年1月至2022年10月在南京医科大学第一附属医院血液科诊断的13例aCLL患者，所有患者均行淋巴结（结外）病灶活检或粗针穿刺经病理诊断为aCLL，诊断标准参照2016年WHO分型。CLL患者初诊时均行外周血流式细胞术免疫表型检测，SLL患者初诊时均行淋巴结穿刺或活检病理检测，诊断标准参照iwCLL2018指南[5]。

2. 临床资料：纳入了包括年龄、性别、既往治疗方案、进展至加速期时间、有无大包块病灶、PET-CT SUVmax、免疫球蛋白重链可变区（IGHV）突变状态、FISH、染色体核型等。部分有条件的患者对诊断aCLL时的骨髓、外周血、病理组织或血浆标本进行了慢性淋巴细胞增生性疾病72分子二代测序（NGS）（上海睿昂基因公司）检测。

3. 疗效评估：对于接受包含化学免疫治疗方案的患者在化疗中期、化疗结束时及化疗结束后每3～6个月进行疗效评估，直至疾病进展（PD）。对于仅接受小分子靶向药物治疗方案的患者每3～6个月进行疗效评估，直至PD。疗效评估项目包括：体格检查、影像学检查、血液学检查和骨髓穿刺/活检，影像学检查包括PET-CT或颈、胸、腹、盆腔增强CT，必要时根据临床指征进行其他相关检查。疗效评估根据iwCLL 2018指南（SLL患者根据Lugano2014标准）评价，包括完全缓解（CR）、部分缓解（PR）、疾病稳定（SD）、PD，总反应率（ORR）为CR率和PR率之和。

4. 随访：本研究随访截止时间为2023年1月27日，中位随访时间14.3（0.3～33.4）个月，无进展生存（PFS）时间定义为自患者确诊aCLL并接受治疗至PD或因任何原因死亡或末次随访的时间，OS时间定义为自患者确诊aCLL至因任何原因死亡或末次随访的时间。

5. 统计学处理：统计分析采用R 4.2.2软件，计数资料用例数表示，计量资料用中位数（范围）表示。应用Kaplan-Meier法绘制生存曲线，使用Cox模型进行生存分析，*P*<0.05为差异有统计学意义。

## 结果

1. 临床特征：本研究纳入了13例aCLL患者，男7例，女6例，诊断aCLL时的中位年龄为54（35～72）岁，除外3例初诊即诊断aCLL的患者，余10例患者自诊断为CLL/SLL发展至aCLL的中位时间为49个月（表1）。5例患者既往未接受治疗，1例患者既往仅接受化学免疫治疗，7例患者既往接受包含布鲁顿酪氨酸激酶抑制剂（BTKi）的治疗，既往接受治疗的中位线数为2。13例患者均因短期内出现淋巴结增大而就诊。诊断aCLL时患者外周血淋巴细胞绝对计数（ALC）中位数为6.7（2.1～180.2）×10^9^/L，分别有6例和3例患者出现HGB降低和PLT降低。血清LDH和β_2_-微球蛋白的中位数分别为271.0（187.0～2 630.6）U/L和3.5（1.9～8.6）mg/L。4例患者伴有B症状，6例患者有大包块病灶（病灶最大径≥5 cm）。3例患者初诊时经穿刺活检诊断aCLL后行PET-CT检查，其余9例患者为除外Richter转化，选取PET-CT代谢摄取最高处/次高处（其中2例患者因代谢摄取最高处病灶无法穿刺活检而取次高处进行病理检查，2例患者代谢摄取最高值分别为5.56和7.67，代谢摄取次高值分别为4.10和4.72）进行淋巴结（结外）病灶活检或粗针穿刺确诊。患者PET-CT的中位SUVmax为6.96（2.51～11.90），10例患者SUVmax在5～10之间，仅1例患者SUVmax>10。与Richter综合征不同，部分aCLL患者病灶内部摄取呈不均匀性，且不同病灶间摄取异质性大。

**表1:** 13例加速期慢性淋巴细胞白血病（aCLL）患者的临床及分子生物学特征、治疗及预后

例号	性别	诊断aCLL时年龄（岁）	发展至aCLL的时间（月）	SUVmax	大包块病灶	IGHV突变状态	复杂核型	TP53缺失或突变	诊断aCLL前的治疗疗程数（既往治疗方案）	治疗方案	PFS时间（月）	最佳疗效
1	男	72	0	5.38	无	有突变	无	无	0	Z-Ben-R	8.8	CR
2	男	53	0	5.56	有	无突变	无	无	0	I-FC-R	29.5	CR
3	女	54	24.4	8.10	有	无突变	无	无	0	I-R-DA-EPOCH	28.4	CR
4	女	52	0	2.51	无	无突变	有	无	0	Vene-R-DA-EPOCH	17.0	CR
5	男	35	19.0	5.77	有	无突变	无	无	0	O-FC-G	6.8	PR
6	男	56	38.9	4.22	无	无突变	NA	NA	2（Chl→FC-R）	Z-R-CHOP	14.3	CR
7	男	63	93.7	6.25	有	有突变	有	无	3（FC-R→Bcl-2i→I）	CAR-T细胞	11.2	PR
8	男	68	44.8	9.02	有	有突变	有	无	2（Chl→I）	NA	0.3	NA
9	男	54	72.9	11.90	无	无突变	无	无	2（Chl→Z）	Vene-G-DA-EPOCH	15.3	CR
10	女	65	42.7	8.82	有	无突变	有	有	2（Ben→Z）	Vene	26.3	PR
11	女	45	60.4	7.67	无	无突变	NA	有	3（Chl→I→Z）	G-DA-EPOCH	7.0	PR
12	女	69	53.1	NA	NA	无突变	无	无	2（Chl→I）	I-Vene	18.3	CR
13	女	51	158.2	9.90	无	无突变	有	无	7（FC-R、FC→COP、CHOPE→Hyper-CVAD→I→Bcl-2i→I-R2-BeGEV→Vene）	BTKi	1.6	PD

注 IGHV：免疫球蛋白重链可变区；Chl：苯丁酸氮芥；FC：氟达拉滨+环磷酰胺；R：利妥昔单抗；Bcl-2i：Bcl-2抑制剂；I：伊布替尼；Z：泽布替尼；Ben：苯达莫司汀；COP：环磷酰胺+长春新碱+泼尼松；CHOPE：环磷酰胺+多柔比星+长春新碱+泼尼松+依托泊苷；Hyper-CVAD：A方案：环磷酰胺+长春新碱+长春新碱+地塞米松；B方案：甲氨蝶呤+阿糖胞苷；R2：利妥昔单抗+来那度胺；BeGEV：苯达莫司汀+吉西他滨+长春瑞滨；Vene：维奈克拉；DA-EPOCH：依托泊苷+泼尼松+长春新碱+环磷酰胺+多柔比星；O：奥布替尼；G：奥妥珠单抗；CAR-T细胞：嵌合抗原受体T细胞；CHOP：环磷酰胺+多柔比星+长春新碱+泼尼松；BTKi：布鲁顿酪氨酸激酶抑制剂；PFS：无进展生存；CR：完全缓解；PR：部分缓解；PD：疾病进展；NA：无数据

2. 细胞及分子生物学特征：本研究患者中10例患者IGHV无突变，IGHV无突变者诊断为aCLL时的中位年龄较IGHV有突变者小（*P*<0.01）。IGHV 4-39片段的使用率最高（4/13，30.8％），其中2例患者属于同型模式8，此外有1例患者使用骨髓标本时检测出两群使用片段不同的IGHV（IGHV 4-34和IGHV 3-7，均有突变）。为区分和明确aCLL和CLL/SLL是否为疾病演变的不同阶段及是否存在克隆非同源性，我们对部分患者不同阶段的样本进行了检测。5例患者在诊断aCLL时使用病理组织标本检测了IGHV突变状态、使用片段和重排模式，其中4例均与同期或CLL/SLL阶段使用骨髓/外周血标本检测的结果一致；1例患者CLL/SLL阶段IGHV使用片段为1-2，而aCLL病理组织标本IGHV使用片段为1-17，其血浆标本中同时检测出上述两群克隆。aCLL诊断时5例患者为复杂染色体核型，进行FISH检测的患者中3例合并+12，各有1例患者检出del（13q14）及del（6q23），未检测出del（17p）及del（11q）。此外，我们对12例患者可获得的病理组织、骨髓或外周血样本进行了NGS检测，突变频率高的基因依次为ATM（6/12，50％）、NOTCH1（6/12，50％）、SF3B1（5/12，41.7％）、BRAF（3/12，25％）、FBXW7（3/12，25％）、KMT2D（3/12，25％）、KRAS（3/12，25％）、XPO1（3/12，25％），2例检测出TP53（2/12，16.7％）突变（图1）。在7例既往接受BTKi后发生aCLL的患者中，各有3例患者检测出BTK突变（3/7，42.9％）和PLCγ2突变（3/7，42.9％），其中1例患者同时检测出BTK突变和PLCγ2突变。诊断aCLL患者的中位基因突变数目为4个，其中既往未接受治疗患者的中位基因突变数目为3个，既往接受BTKi患者的中位基因突变数目为5个。此外，伴KRAS突变的aCLL患者治疗后中位PFS时间较不伴KRAS突变的患者显著缩短（7.0个月对26.3个月，*P*＝0.015）。

**图1:**
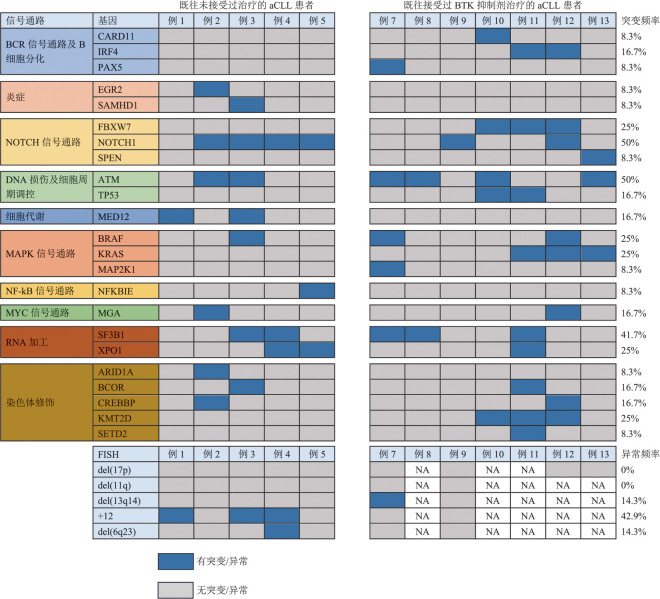
12例患者诊断加速期慢性淋巴细胞白血病（aCLL）时的分子突变类型及FISH结果 例6数据缺失；例1、4、7、8使用骨髓/外周血标本进行二代测序检测，其余8例患者同时使用骨髓/外周血标本和病理组织/血浆标本进行二代测序检测；FISH均使用骨髓标本进行；NA：无数据

3. 治疗及疗效：1例患者失访，12例患者在诊断aCLL后均接受小分子靶向药物和（或）化学免疫治疗，ORR为91.7％，CR率为58.3％。中位随访14.3个月，中位OS时间尚未达到，中位PFS时间为18.9个月。5例后续PD的患者中2例发生Richter转化（例4、例10）。7例患者接受小分子靶向药物联合化学免疫治疗，ORR为100％，CR率为85.7％，其中包括5例既往未治疗的aCLL患者。1例患者获得CR后进行了自体造血干细胞移植，但在7个月后出现进行性ALC增高及PLT下降、淋巴结增大而考虑PD并经病理证实为Richter转化，其余患者随访至今仍处于持续缓解状态。5例既往接受治疗后病情进展的aCLL患者中，3例患者接受小分子靶向药物治疗，1例患者接受化学免疫治疗，均出现PD，中位PFS时间为13个月。仅1例患者接受CD20单抗治疗后进行了CAR-T细胞治疗，目前处于持续缓解状态。

## 讨论

尽管在新药时代下，BTKi、Bcl-2抑制剂等小分子靶向药物已经极大地改变了CLL/SLL患者的治疗策略和预后，但仍然有部分CLL/SLL患者在疾病病程中发生组织学转化或进展，其病程呈侵袭性，预后较CLL/SLL患者差[6]–[11]。目前已有新药时代下Richter转化的临床特征、分子机制及治疗的报道，但尚无aCLL相关研究。

本中心13例aCLL患者的中位年龄为54岁，提示年轻CLL/SLL患者更易发生组织学进展。当CLL/SLL患者病程中出现B症状加重、淋巴结迅速增大、LDH升高及可疑的结外病灶等临床表现怀疑发生Richter转化时，推荐行PET-CT检查选择代谢摄取最高处行穿刺活检及病理检查[12]。本研究纳入的患者除3例初诊即诊断aCLL外，其余患者均为病情进展临床怀疑发生Richter转化才进行病理检查。既往研究将临床怀疑Richter转化患者的SUVmax最佳临界值设为5或10[3],[13]–[14]。近年来，随着越来越多小分子靶向药物的应用，发生Richter转化患者PET-CT SUVmax最佳临界值的敏感度及特异性均不理想，小分子靶向药物的使用有可能降低了以SUVmax＝10作为区分Richter转化最佳截取值的敏感度[15]。Wang等[16]及Kittai等[15]的研究报道，接受小分子靶向药物治疗后发生Richter转化的中位SUVmax为11.3和15.2[15]–[16]。本研究中有12例患者完善了PET-CT检查，中位SUVmax为6.96（2.51～11.9）。aCLL患者的SUVmax较Richter患者更低，为在新药时代下PET-CT识别及区分aCLL和Richter转化患者带来了困难。aCLL诊断的金标准仍为组织病理检查，我中心aCLL患者的病理标本绝大多数为粗针穿刺获得，与活检病理标本相比，存在组织较少、细胞压缩破坏等问题，为疾病的诊断带来了挑战。结合本研究中PET-CT检查的病灶异质性，在穿刺过程中有可能并未真正取到代谢摄取值最高的病灶部位，因此我们建议有条件的中心对于临床怀疑疾病进展且SUVmax≥5的患者在代谢摄取最高处行活检以明确病理诊断。

IGHV突变状态及使用片段被认为是CLL/SLL的一项重要预后因素，IGHV无突变的患者预后不良，且IGHV 4-39使用片段及同型模式8患者发生Richter转化的风险高。Richter转化为弥漫大B细胞淋巴瘤患者根据转化组织IGHV使用片段和重排模式是否与CLL/SLL一致可分为同源性转化和非同源性转化两类，其中同源性转化患者的病程呈高度侵袭性[12],[17]–[18]。本研究中10例患者IGHV无突变，IGHV 4-39片段的使用率高（4/13，30.8％），且其中2例属于同型模式8；5例使用aCLL病理组织检测IGHV使用片段和重排模式的患者中4例与其CLL/SLL阶段一致，1例患者aCLL病理组织与CLL/SLL阶段的IGHV使用片段不同，提示非同源性。但目前缺乏对aCLL患者IGHV突变状态和使用片段的大样本研究报道，其在aCLL中的临床意义尚不明确。+12被认为与CLL/SLL患者不典型的细胞形态相关，其与NOTCH1突变同时存在被认为是CLL/SLL的不良预后因素[19]–[20]。在本研究中42.9％（3/7）的患者检出+12，其中2例合并NOTCH1突变，与既往报道的结论一致。本研究中aCLL患者的NGS结果显示ATM（50％）、NOTCH1（50％）为突变频率最高的基因。既往报道，伴ATM突变的CLL/SLL患者更年轻，在诊断时通常以淋巴结肿大为主要临床表现，至首次治疗时间及OS时间更短，而NOTCH1突变被认为与不良预后以及Richter转化相关[20]–[22]，与本研究中aCLL患者的临床特征符合。此外，部分接受BTKi的患者检测出BTK及PLCγ2突变，这些基因突变被认为与BTKi的耐药相关[23]–[24]。尽管如此，目前仍不能明确ATM、NOTCH1及BTK耐药突变等在aCLL发生发展过程中是否有驱动作用，后续有待进一步探索分析。

尽管aCLL患者的预后较CLL/SLL差，但目前对于aCLL患者的治疗尚无明确推荐，仍然参照CLL/SLL的治疗策略。既往研究报道aCLL患者的预后更接近Richter转化患者，参考Richter转化患者的治疗方案（R-CHOP、R-DA-EPOCH等）[25]–[26]，结合本研究aCLL患者中位诊断年龄较小、大多伴随不良预后因素（如IGHV无突变），我们选择了小分子靶向药物联合化学免疫治疗方案。7例接受联合治疗方案的患者均达到疾病客观缓解（ORR为100％），其中6例达到CR，除1例后续发生病情进展外，其余患者目前均仍处于持续缓解状态。在仅接受单纯小分子靶向药物治疗或单纯化学免疫治疗的4例患者中，仅1例达到CR，且所有患者在后续均发生了PD。提示小分子靶向药物联合化学免疫治疗可能对aCLL患者有更为持久的疗效。尽管如此，1例接受小分子靶向药物联合化学免疫治疗且后续进行自体造血干细胞移植的患者仍发生了PD并确诊为Richter转化（例4），该例患者IGHV无突变，属于同型模式8，且存在复杂染色体核型、+12及NOTCH1突变，属于Richter转化的高风险患者。提示对于此类患者，接受联合治疗及自体造血干细胞移植后可能仍需要行CAR-T细胞治疗或小分子靶向药物维持治疗以达到更深度的缓解，延长疾病缓解时间。此外，本研究显示伴KRAS突变的aCLL患者经治疗后中位PFS时间较无突变患者显著缩短，提示对于这部分伴KRAS突变的aCLL患者仍需要探索有效的治疗方案。

尽管既往报道及本研究中aCLL患者临床呈侵袭性且预后不佳，但其并不等同于Richter转化。aCLL在病理上以扩张/融合的增殖中心和高增殖活性为特点，高倍镜下增殖中心可见大的副免疫母细胞和幼淋巴细胞，而并非Richter转化中所见的弥漫分布的中心母细胞和免疫母细胞，在诊断时需仔细鉴别。在临床表现上，aCLL患者与Richter转化相似，可出现进行性淋巴结增大、HGB及PLT进行性下降、血清LDH升高以及B症状等，CLL/SLL患者出现上述症状时除Richter转化外还需警惕aCLL发生。在本研究中aCLL患者合并多种预后不良因素，同时也包括Richter转化的危险因素，如IGHV无突变、IGHV 4-39片段使用和同型模式8、NOTCH1突变等，但合并TP53缺失或突变患者的比例较Richter转化低。本研究中2例aCLL患者在后续病程中出现病情进展，确诊为Richter转化，但aCLL与Richter转化的相关性尚不明确。

综上所述，本研究回顾性分析了13例aCLL患者的临床特征及预后。无论既往是否接受治疗，对于出现疾病进展特别是出现大包块病灶的CLL/SLL患者需警惕aCLL的发生，建议此类患者行PET-CT检查，SUVmax≥5的患者在代谢摄取最高处行活检明确病理诊断。本中心大部分aCLL患者为IGHV无突变状态，+12及ATM、NOTCH1突变比例高，伴KRAS突变的患者预后不佳。aCLL目前治疗仍参考CLL/SLL，对于部分靶向药物耐药同时合并多种不良预后因素特别是转化高风险人群可能需要接受联合化疗、CAR-T细胞治疗等以达到更深度的缓解，其发生发展的具体机制及治疗、预后有待进一步探索。

## References

[1] Swerdlow SH, Campo E, Pileri SA (2016). The 2016 revision of the World Health Organization classification of lymphoid neoplasms[J]. Blood.

[2] Giné E, Martinez A, Villamor N (2010). Expanded and highly active proliferation centers identify a histological subtype of chronic lymphocytic leukemia (“accelerated” chronic lymphocytic leukemia) with aggressive clinical behavior[J]. Haematologica.

[3] Falchi L, Keating MJ, Marom EM (2014). Correlation between FDG/PET, histology, characteristics, and survival in 332 patients with chronic lymphoid leukemia[J]. Blood.

[4] Ciccone M, Agostinelli C, Rigolin GM (2012). Proliferation centers in chronic lymphocytic leukemia: correlation with cytogenetic and clinicobiological features in consecutive patients analyzed on tissue microarrays[J]. Leukemia.

[5] Hallek M, Cheson BD, Catovsky D (2018). iwCLL guidelines for diagnosis, indications for treatment, response assessment, and supportive management of CLL[J]. Blood.

[6] Burger JA, Tedeschi A, Barr PM (2015). Ibrutinib as Initial Therapy for Patients with Chronic Lymphocytic Leukemia[J]. N Engl J Med.

[7] Woyach JA, Ruppert AS, Heerema NA (2018). Ibrutinib Regimens versus Chemoimmunotherapy in Older Patients with Untreated CLL[J]. N Engl J Med.

[8] Fischer K, Al-Sawaf O, Bahlo J (2019). Venetoclax and Obinutuzumab in Patients with CLL and Coexisting Conditions[J]. N Engl J Med.

[9] Maddocks KJ, Ruppert AS, Lozanski G (2015). Etiology of Ibrutinib Therapy Discontinuation and Outcomes in Patients With Chronic Lymphocytic Leukemia[J]. JAMA Oncol.

[10] Hampel PJ, Rabe KG, Call TG (2022). Clinical outcomes in patients with chronic lymphocytic leukemia with disease progression on ibrutinib[J]. Blood Cancer J.

[11] Anderson MA, Tam C, Lew TE (2017). Clinicopathological features and outcomes of progression of CLL on the BCL2 inhibitor venetoclax[J]. Blood.

[12] Petrackova A, Turcsanyi P, Papajik T (2021). Revisiting Richter transformation in the era of novel CLL agents[J]. Blood Rev.

[13] Mauro FR, Chauvie S, Paoloni F (2015). Diagnostic and prognostic role of PET/CT in patients with chronic lymphocytic leukemia and progressive disease[J]. Leukemia.

[14] Michallet AS, Sesques P, Rabe KG (2016). An 18F-FDG-PET maximum standardized uptake value > 10 represents a novel valid marker for discerning Richter's Syndrome[J]. Leuk Lymphoma.

[15] Kittai AS, Huang Y, Beckwith KA (2023). Patient characteristics that predict Richter's transformation in patients with chronic lymphocytic leukemia treated with ibrutinib[J]. Am J Hematol.

[16] Wang Y, Rabe KG, Bold MS (2020). The role of 18F-FDG-PET in detecting Richter's transformation of chronic lymphocytic leukemia in patients receiving therapy with a B-cell receptor inhibitor[J]. Haematologica.

[17] Rossi D, Spina V, Cerri M (2009). Stereotyped B-cell receptor is an independent risk factor of chronic lymphocytic leukemia transformation to Richter syndrome[J]. Clin Cancer Res.

[18] Rossi D, Spina V, Bomben R (2013). Association between molecular lesions and specific B-cell receptor subsets in chronic lymphocytic leukemia[J]. Blood.

[19] Del Giudice I, Rossi D, Chiaretti S (2012). NOTCH1 mutations in +12 chronic lymphocytic leukemia (CLL) confer an unfavorable prognosis, induce a distinctive transcriptional profiling and refine the intermediate prognosis of +12 CLL[J]. Haematologica.

[20] Nadeu F, Delgado J, Royo C (2016). Clinical impact of clonal and subclonal TP53, SF3B1, BIRC3, NOTCH1, and ATM mutations in chronic lymphocytic leukemia[J]. Blood.

[21] Fabbri G, Rasi S, Rossi D (2011). Analysis of the chronic lymphocytic leukemia coding genome: role of NOTCH1 mutational activation[J]. J Exp Med.

[22] Fabbri G, Khiabanian H, Holmes AB (2013). Genetic lesions associated with chronic lymphocytic leukemia transformation to Richter syndrome[J]. J Exp Med.

[23] Woyach JA, Furman RR, Liu TM (2014). Resistance mechanisms for the Bruton's tyrosine kinase inhibitor ibrutinib[J]. N Engl J Med.

[24] Bödör C, Kotmayer L, László T (2021). Screening and monitoring of the BTK(C481S) mutation in a real-world cohort of patients with relapsed/refractory chronic lymphocytic leukaemia during ibrutinib therapy[J]. Br J Haematol.

[25] Langerbeins P, Busch R, Anheier N (2014). Poor efficacy and tolerability of R-CHOP in relapsed/refractory chronic lymphocytic leukemia and Richter transformation[J]. Am J Hematol.

[26] Davids MS, Rogers KA, Tyekucheva S (2022). Venetoclax plus dose-adjusted R-EPOCH for Richter syndrome[J]. Blood.

